# Costs of conservative management of early-stage prostate cancer compared to radical prostatectomy–a claims data analysis

**DOI:** 10.1186/s12913-016-1886-4

**Published:** 2016-11-18

**Authors:** Alina Brandes, Florian Koerber, Larissa Schwarzkopf, Matthias Hunger, Wolf H. Rogowski, Raphaela Waidelich

**Affiliations:** 1 Institute of Health Economics and Health Care Management, Helmholtz Zentrum München, Neuherberg, Germany; 2 Institute of Public Health and Nursing Research, Health Sciences, University of Bremen, Bremen, Germany; 3 Department of Urology, Ludwig-Maximilian University, Munich, Germany

**Keywords:** Prostate cancer, Cost and cost analysis, Radical prostatectomy, Conservative management

## Abstract

**Background:**

Due to widespread PSA testing incidence rates of localized prostate cancer increase but curative treatment is often not required. Overtreatment imposes a substantial economic burden on health care systems. We compared the direct medical costs of conservative management and radical therapy for the management of early-stage prostate cancer in routine care.

**Methods:**

An observational study design is chosen based on claims data of a German statutory health insurance fund for the years 2008–2011. Three hundred fifty-three age-matched men diagnosed with prostate cancer and treated with conservative management and radical prostatectomy, are included. Individuals with diagnoses of metastases or treatment of advanced prostate cancer are excluded.

In an excess cost approach direct medical costs are considered from an insured community perspective for in- and outpatient care, pharmaceuticals, physiotherapy, and assistive technologies. Generalized linear models adjust for comorbidity by Charlson comorbidity score and recycled predictions method calculates per capita costs per treatment strategy.

**Results:**

After follow-up of 2.5 years per capita costs of conservative management are €6611 lower than costs of prostatectomy ([−9734;−3547], *p* < 0.0001). Complications increase costs of assistive technologies by 30% (*p* = 0.0182), but do not influence any other costs. Results are robust to cost outliers and incidence of prostate cancer diagnosis. The short time horizon does not allow assessing long-term consequences of conservative management.

**Conclusions:**

At a time horizon of 2.5 years, conservative management is preferable to radical prostatectomy in terms of costs. Claims data analysis is limited in the selection of comparable treatment groups, as clinical information is scarce and bias due to non-randomization can only be partly mitigated by matching and confounder adjustment.

**Electronic supplementary material:**

The online version of this article (doi:10.1186/s12913-016-1886-4) contains supplementary material, which is available to authorized users.

## Background

Prostate cancer (PCa) is the second most common cancer in men worldwide and the most common in Germany [1, 2]. Annual health care spending due to PCa is substantial and accounts for €5.43 billion in the European Union (EU). Germany exhibits the highest PCa-related health care costs per person in the EU, mostly due to inpatient expenditures [3]. Due to wide-spread prostate specific antigen (PSA) testing incidence rates and health care spending will increase significantly in the EU in the future. Detected tumors are often localized, clinically insignificant cancers, though. Data suggest that many men with localized PCa do not benefit from curative treatment with radical prostatectomy (RP) in terms of survival because tumor progression is so slow that no treatment is required [4].

The conservative management (CM) strategies active surveillance (AS) and watchful waiting (WW) are proposed in this context for the treatment of localized PCa to reduce overtreatment and subsequent complications such as erectile dysfunction (ED) and urinary incontinence (IC). Under AS regular biopsies, PSA tests, and digital rectal examinations are performed to initiate curative treatment if tumor progression occurs. If life expectancy is less than 10 years or comorbidity does not allow any other form of PCa-treatment WW is recommended. This strategy has no standardized follow-up scheme; symptom-oriented, palliative therapy is initiated if disease progresses [5]. A CM-strategy may save health care costs of unnecessary curative therapy and treatment of its adverse effects.

Current economic studies suggest that, while health outcome in terms of survival and quality of life is similar for CM and curative treatment, CM is a cost-saving strategy over the first 5 to 10 years of treatment [6–12]. Most studies base cost analysis on US reimbursement values, which are often not representative for European health care [13]. To guide treatment decisions in a European health care context observational studies are needed displaying routine care and actual health care spending on CM compared to RP.

In a German health care context, claims data of statutory health insurance (SHI) funds are a valuable evidence source as medical care, resource use, and costs are documented in detail and over a long period of time for a large cohort of patients. About 85% of the German population is insured within the social security system of SHI, which is characterized by pay-as-you-go financing and income-dependent insurance contributions. The remaining 15% are covered by private insurance. SHI covers inpatient and outpatient care, pharmaceuticals, physiotherapy, and assistive technologies. Co-payments of patients are compulsory, especially for pharmaceuticals and medical aids [14].

It is the objective of this paper to analyze claims data of a German AOK SHI fund regarding costs of CM compared to RP in an age-matched and comorbidity-adjusted cohort of men with early-stage PCa.

## Methods

### Data

AOK Baden-Württemberg is the largest SHI fund in the south-western German federal state of Baden-Württemberg with about 3.8 million insured individuals. About 25% of the insured population is retired and over 65 years. Data on all claims incurred at AOK between 2008 and 2011 were provided on a patient level. Co-payments to medical services covered by SHI are included in the dataset, whereas patients’ out-of-pocket payments for other services are not. German data protection laws were considered during extraction and analysis of data and AOK approved of the intended use of the data. An ethics committee was consulted regarding this study; ethics approval is not necessary as identification of individuals is not possible in the dataset.

### Study design and cohort selection

A prospective, longitudinal study design is chosen, where a cohort of men diagnosed with early-stage PCa is followed from the point of treatment initiation.

**Fig. 1:**
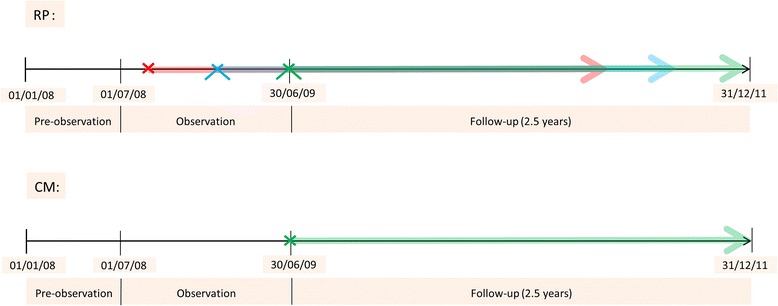
Study timeline

The study period is January 1st 2008 to December 31st 2011 and is divided into three sections (Fig. 1):

1. The 6-month pre-observation period (January 1st 2008 to June 30th 2008) is created as a basis for the calculation of the Charlson comorbidity score (CCS), in order to allow for comorbidity adjustment of costs.
2. In the 12-month observation period from July 1st 2008 to June 30th 2009 PCa-cases are identified and categorized into the two treatment-groups CM and RP.
3. Both treatment groups are followed-up for a period of exactly 2.5 years (follow-up period). In case of RP, follow-up time starts individually for each person after the date of the initial prostatectomy procedure and follows each man for 2.5 years on an individual basis. Men under CM, on the other hand, are followed for a fixed period from July 1st 2009 to December 31st 2011. The reason for this is, firstly, that the starting point of CM cannot be established in the cohort; an artificial starting point has to be created, which is the beginning of the observation period (July 1st 2008). Secondly, follow-up is not intended to start with the onset of CM. This is due to the cohort selection, where men under CM have to be surveyed for at least 12 months (the observation period) to be included in the cohort; men dying in this period are not considered for analysis. In the RP- group a single event in the observation period determines inclusion in the cohort, which in turn possibly includes men dying in this period. To account for this bias, follow-up of AS is offset by 12 months.

**Fig. 2:**
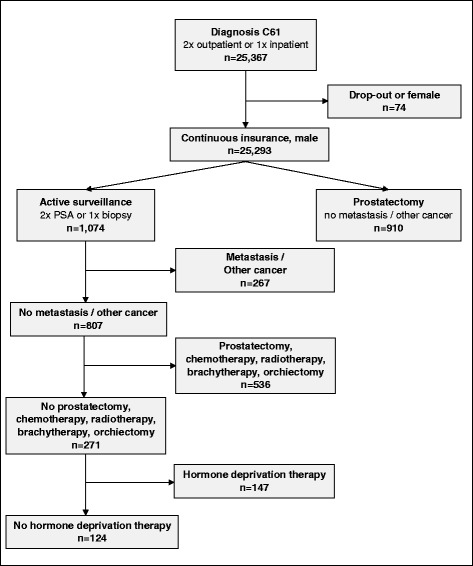
Cohort selection

The baseline dataset includes all men with an ICD-10 diagnosis ‘C61–Malignant neoplasm of prostate’ in the 12-month observation period. PCa diagnosis is validated by only considering men with at least one inpatient or at least two outpatient diagnostic codes (*N* = 25,367). After exclusion of 71 individuals not constantly insured at AOK Baden-Württemberg in the study period (defined by the coding of ‘transition to another insurance fund’ in the claims data) and three individuals with female gender coding, the baseline data set includes 25,293 individuals (Fig. 2, Additional file 1: Table S1 and Table S2).

CM is defined by outpatient procedure codes of at least two PSA tests and one prostate biopsy during the observation period, including AS- and WW-patients [15]. Additionally, patients undergoing any form of PCa-specific therapy other than CM are excluded to avoid misclassification of patients due for curative treatment as CM cases. In the follow-up period, men in the CM-group do not have to be surveyed as defined in the observation period; they may move on to any other form of PCa-specific therapy.

RP is defined by procedure codes on open, laparoscopic, or robotic-assisted radical prostatectomy. Patients with metastatic disease or any other cancer disease coding are excluded. Exclusion of any PCa-specific therapy other than RP is not necessary, as these are not found in the RP-group.

Considering the inclusion criteria the cohort consists of 124 and 910 individuals under CM and RP, respectively.

### Statistical analysis

To control for age as a confounder of cost differences, treatment groups are matched by +/− 2 years. After matching in a ratio of 1:2 the CM-group includes 107 individuals, and the RP-group includes 214 individuals.

Patients’ age and CCS as well as prevalence of ED, IC, and benign prostate hyperplasia (BPH) are calculated at baseline before and after matching. For calculation of CCS the comorbidity-group ‘cancer’ is set 0, because diseases other than PCa are of interest for adjustment.

We use an excess cost approach to display cost differences between treatment strategies for inpatient and outpatient care, pharmaceuticals, physiotherapy, and assistive technologies. All direct medical costs and co-payments are considered that are relevant for the perspective of the SHI scheme insured community (§ 35b (1) SGBV) [16]. Comorbidity-adjusted cost differences between treatment groups are estimated by a generalized linear model (GLM) with a gamma distribution and log link to account for the typically skewed distribution of cost data. If less than 10% of individuals in the cohort have zero costs a small amount of 1 Euro (€) is assigned to include them in the analysis [17]. If individuals with zero costs account for more than 10% of the cohort a two-part model is used: at first, the probability of health care expenditure is predicted with a logistic regression model. Secondly, costs are estimated by a GLM, as described above, conditional for nonzero costs. To derive unconditional costs the probability of expenditure is multiplied by the predicted conditional costs [18]. Recycled predictions are used to estimate per capita costs per treatment strategy in addition to percentage values of differences reported by the GLM [19]. All costs are rounded to the nearest € to present only full € amounts with no cent values; discounting is not considered due to the short study period.

Ninety-five percent confidence intervals (CI) are calculated for costs via a non-parametric bootstrap approach based on 1000 replications. Difference in costs is tested via bootstrap hypothesis testing and *p*-values less or equal than 0.05 are considered statistically significant [20]. The CCS is included in the regression models as a continuous variable [21, 22]. To additionally estimate the influence of ED and IC on treatment costs, complication is included in the regression models as a binary variable. Extended models with an interaction between treatment strategy and CCS do not improve model fit.

In a sensitivity analysis we include only incident PCa cases, with no C61 code in the pre-observation period–assuming that CM is initiated with the first PCa-diagnosis–to estimate the influence of the cohort selection on outcomes.

Statistical analyses are performed with the software package SAS, version 9.3.

## Results

### Descriptive analysis

Mean age at baseline after matching is 69 years (STD 6.80) in the RP-group and 70 years (STD 7.13) in the CM-group, with a mean CCS of 0.11 and 0.19, respectively. ED, IC and BPH are most prevalent in the RP-group at baseline (Table 1).

**Table 1:** Baseline characteristics before and after matching

	Before matching		After matching	
	RP	CM	RP	CM
Total (n)	910	124	214	107
Age (mean, STD)	66 (6.64)	70 (8.31)	69 (6.80)	70 (7.13)
CCS (mean, STD)	0.13 (0.71)	0.19 (0.62)	0.11 (0.63)	0.19 (0.63)
ED (n, prop.)	79 (0.09)	5 (0.04)	24 (0.11)	5 (0.05)
IC (n, prop.)	18 (0.02)	5 (0.04)	10 (0.05)	3 (0.03)
BPH (n, prop.)	511 (0.56)	83 (0.67)	164 (0.77)	73 (0.68)

*CM* conservative management, *n* number, *prop.* proportion, *RP* radical prostatectomy, *STD* standard deviation

### Cost analysis

**Fig. 3:**
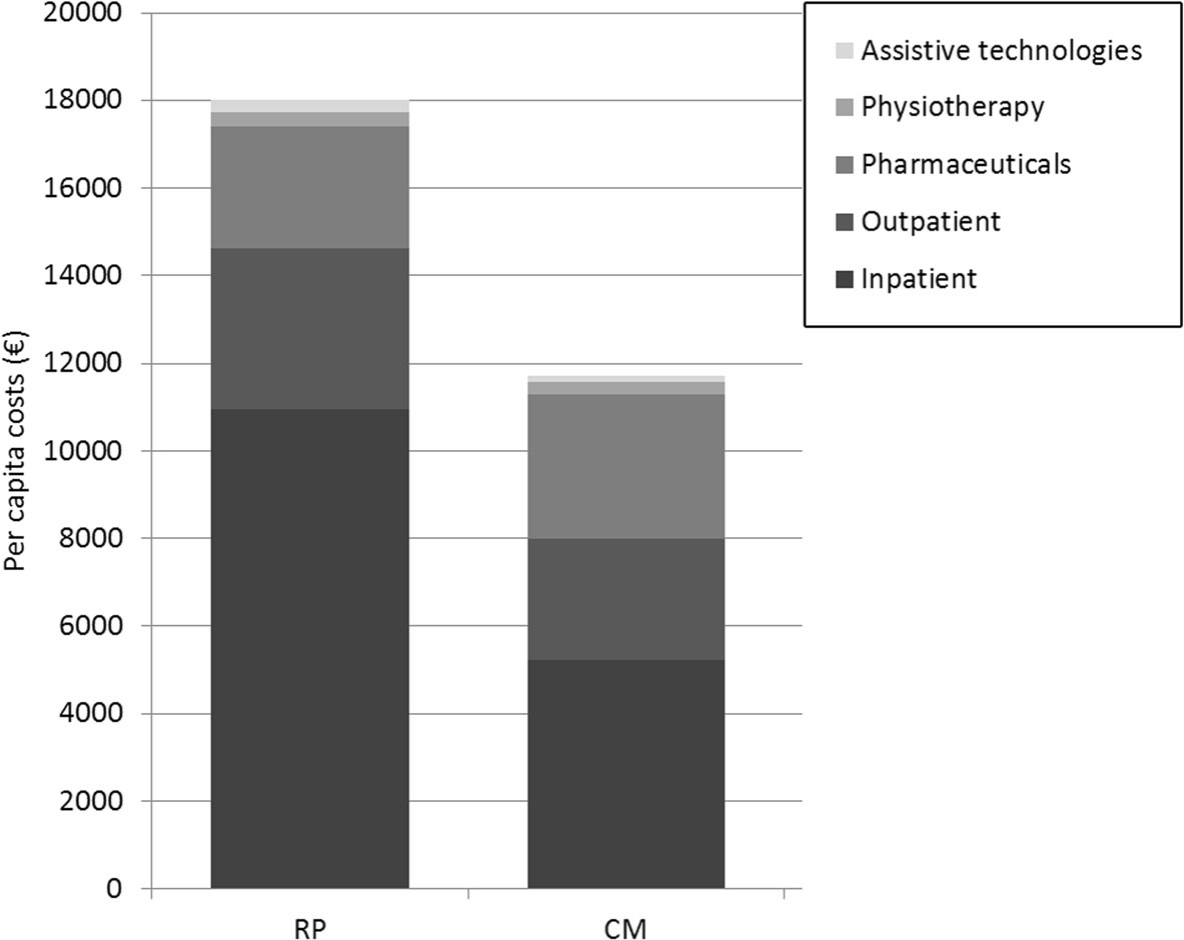
Costs per capita (€)–unadjusted

Figure 3 shows unadjusted per capita costs for the two treatment strategies grouped by inpatient and outpatient treatment, pharmaceuticals, physiotherapy, and assistive technologies. RP has overall higher mean costs, when compared to CM. Comorbidity-adjusted analysis confirms this (RP: €18,544, CM: €11,933).

**Fig. 4:**
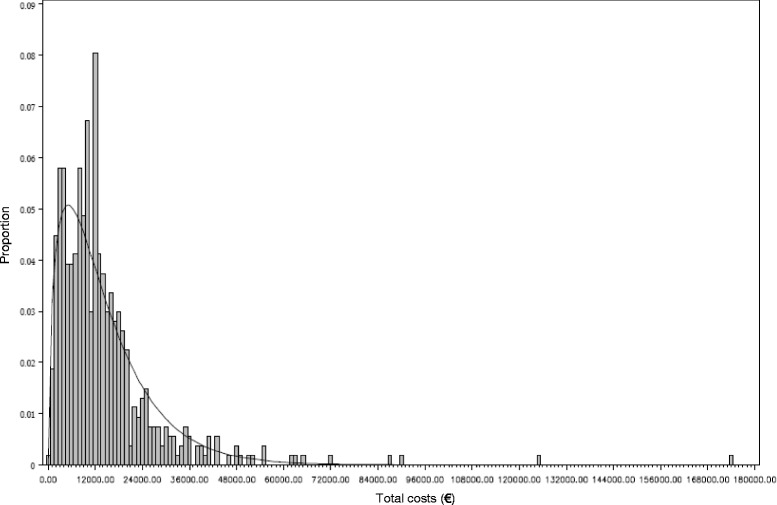
Histogram total costs

Comparison of adjusted costs (Table 2) displays that CM has significantly lower mean inpatient (€−5845 [−7632;−3895], *p* < 0.0001) and outpatient costs (€−961 [−1622;−361], *p* = 0.002) as well as costs for assistive technologies (€−141 [−230;−50], *p* = 0.006) than RP. Overall, total incremental costs of CM are significantly lower compared to RP (€−6611 [−9734;−3547], *p* < 0.0001) (Fig. 4). Inclusion of complication as a binary variable in the regression models does not change cost differences between treatment strategies. Concerning total costs there is no significant cost difference found between individuals with and without complications. In case of assistive technologies a significant 30% increase in costs for individuals with complications is estimated (*p* = 0.018).

**Table 2:** Difference in costs (€) between CM and RP-adjusted

	CM–RP		
	Mean	95% CI	*P*-value
Inpatient	−5845	−7632 to −3895	<0.0001
Outpatient	−961	−1622 to −361	0.002
Pharmaceuticals	587	−556 to 1718	0.274
Physiotherapy	−58	−214 to 114	0.460
Assistive technologies	−141	−230 to −50	0.006
Total costs	−6611	−9734 to −3547	<0.0001

*CM* conservative management, *RP* radical prostatectomy

For the analysis of inpatient, outpatient, pharmaceutical, physiotherapy, and assistive technology costs two-part models are used, as more than 10% of individuals have zero costs respectively. Total costs are analyzed in a one-part model, as no individuals have zero costs.

### Sensitivity analysis

Analysis of costs is repeated including incident PCa-cases only, which comprised of an age-matched cohort of 320 men (CM: 64, RP: 128). Cost analysis reveals that mean comorbidity-adjusted total costs of CM increase by €1496 to €13,430 in total compared to base case. Outpatient costs of CM also increase compared to base case from €2332 to €2499. Overall, CM is still significantly less costly than RP (€−5263 [−9530;−1164], *p* = 0.016) in the incident cohort.

## Discussion

This is, to our knowledge, the first observational study comparing costs of CM to RP in a European health care context. Use of claims data allows picturing actual treatment practice of early-stage PCa in Germany. Cost analysis is based on exact and detailed cost information on different health care sectors which is representative for the costs incurred by the SHI scheme insured community, contrary to many clinical trials. Compared to modeling studies assumptions on resource use and reimbursement practice are not required.

CM is overall significantly less costly than RP which mainly originates in the significant difference of inpatient costs, presumably due to the high costs of the initial RP-surgery. Compared to RP outpatient care is also less costly under CM in the period of 2.5 years follow-up, despite that the main costs of CM arise in outpatient care. This is due to different cost patterns, where surgery incurs high initial outpatient costs (e.g. for post-operative care after RP) while costs of surveillance, especially AS, are more equally distributed over time [6–9]. The sensitivity analysis with incident PCa-cases–which assumes that CM is initiated with the first PCa-diagnosis–shows that outpatient costs increase compared to base case which may lead to the conclusion that CM-costs are comparably higher in the initial phase.

In the base case analysis pharmaceutical costs are higher under CM compared to RP, not significantly though; this may be explained by pharmaceutical treatment of BPH in the CM-group which is not necessary in the RP-group.

Published cost studies with a European health care context comparing CM and RP report similar cost differences to our study. Andersson et al. [23] report €6123 lower costs of WW compared to RP over a follow-up of 12 years based on data from the SPCG-4 clinical trial [23]. In a lifetime modeling study, Koerber et al. [7] estimate that under AS compared to RP costs are reduced by about €6900 [7]. Both in the published studies and in our study, the cost difference between CM and RP is almost exclusively due to the costs of the initial RP-surgery.

Several US-based observational and modeling studies show that CM is the least costly strategy over the whole study duration, consistent with the results presented here [6, 10, 12, 8, 24]. Only the study by Perlroth et al. [11] reports that from year 2 of the study on costs of CM become equal to costs of RP [11]. Perlroth et al. do not state unit costs of the surveillance scheme; however, other US-based studies show that the unit costs of prostate biopsy ($605–$1102) alone are considerably higher in the US health-care context than unit costs of the whole surveillance scheme (PSA testing and biopsy) in Germany (€44) [6, 8, 24]. Because of the limited study duration only short-term costs can be assessed in this study; as published studies do, however, suggest that cost differences between CM and curative treatment, especially RP, arise in the first years after treatment, the results of this study may support the conclusion that costs of CM do not arise to costs of RP in a lifetime perspective [25].

Our study has some limitations. Regarding cohort selection, there is no information on tumor stage or Gleason score included in claims data, which might allow clinical classification of PCa. To account for this limitation we define early-stage PCa as absence of both diagnoses of metastases and treatment associated with recurrence or advanced tumor progression. Inclusion in the CM-group cannot be based on clinical criteria or specific procedure codes and therefore a distinction between WW- and AS-patients is not possible. Seven Percent of men under CM receive at least one biopsy during follow-up which suggests that these are under AS, while the remaining men may be under WW. The cohort’s life expectancy, however, is with a mean age of 70 years at baseline greater than 10 years and men are–according to treatment guideline–not eligible to WW. Patients might actually be under AS, but are not surveyed by regular biopsies according to guideline. This might be due to current studies on adverse effects of serial biopsies on erectile function and infectious complications [26, 27]. Furthermore, randomization of individuals into treatment groups is not possible as in any other observational study; estimated differences between groups might be attributed to unequal distribution of confounding variables. This bias is reduced by matching and regression analysis only to a certain degree, because the number of variables available for confounder adjustment is limited in our data set. Also, descriptive analysis of patient characteristics after matching shows that men in the CM-group have more co-morbidities than in the RP-group (CCS 0.19 vs. 0.11), which is adjusted for in regression analysis. Men in the RP-group, on the other hand, show higher rates of BPH than men under CM, which might be explained by the treatment itself; radical prostatectomy is a treatment for BPH, too.

Regarding the study design, a 12-month observation period is chosen which excludes CM-patients who receive curative therapy in this period. The probability for curative treatment in the first year of CM is, however, low (<5%) [28]. Also it is necessary in this study to allow enough time to confirm the CM-strategy and exclude patients who are waiting for curative treatment, which is done 6 months after the initial PCa-diagnosis latest [29]. This study might underestimate costs of the CM-strategy slightly by the exclusion of cases with curative treatment in the first year. Alternative treatment options like radiotherapy were excluded from the study due to the availability of claims data for a limited duration. Complications after radiotherapy, usually develop after a longer period than analyzed here, which might underestimate the costs of radiotherapy.

Regarding cost analysis, calculation of costs for single procedures is not conducted in this study as case numbers are too small to be representative. CCS is not able to adjust for comorbidities with high outpatient and pharmaceutical costs as the score was developed to assess inpatient mortality. We do not account for dependency of data due to matching by using conditional regression analysis. Dependency of data is very low as we only match for age; also no longitudinal analysis of single individuals is intended.

Generalizability of results to the general population is limited as claims data cannot report out-of-pocket-payments. Also, due to the excess cost approach absolute costs do not reflect PCa treatment specific costs and no conclusion can be inferred on the costs of RP and CM from this study.

## Conclusions

Overall, our analysis indicates that initial treatment costs as well as short-term follow-up costs of CM based on claims data of a German SHI fund are significantly lower than the costs of curative therapy with RP for early-stage PCa–predominantly due to the high initial costs of the surgery. Treatment of complications following initial therapy has a very small impact on costs. These results must be interpreted in light of the limitations regarding cohort selection in claims data.

Further research is necessary to analyze costs of CM compared to curative treatment over a longer time horizon than reported in this study including costs of long-term complications and end-of-life care.

## Additional file

Additional file 1: Table S1. Diagnostic codes for cohort selection. **Table S2.** Procedure codes for cohort selection. (DOC 34 kb)

## Abbreviations

AS: Active surveillance
BPH: Benign prostate hyperplasia
CCS: Charlson comorbidity score
CI: Confidence intervals
CM: Conservative management
ED: Erectile dysfunction
EU: European Union
GLM: Generalized linear model
IC: Urinary incontinence
PCa: Prostate cancer
PSA: Prostate specific antigen
RP: Radical prostatectomy
SHI: Statutory health insurance
WW: Watchful waiting
