# Supplementary material for: Costs of conservative management of early-stage prostate cancer compared to radical prostatectomy–a claims data analysis
Source: BMC Health Serv Res. 2016 Nov 18;16:664. doi: 10.1186/s12913-016-1886-4 (PMC5116165; doi:10.1186/s12913-016-1886-4)
Supplement: Additional file 1: Table S1. — Diagnostic codes for cohort selection. Table S2. Procedure codes for cohort selection. (DOC 34 kb) [file 12913_2016_1886_MOESM1_ESM.doc]

# Supplementary file

**Table S1** Diagnostic codes for cohort selection.

| Diagnosis | ICD-10 code |
| --- | --- |
| Prostate cancer | C61 |
| Erectile dysfunction | N48.4, F52.2 |
| Urinary incontinence | N39.3, N39.4, R32, F98.0 |
| Benign prostate hyperplasia | N40 |

**Table S2 Procedure codes for cohort selection.**

| Procedure | Inpatient (OPS) | Outpatient (EBM) | Pharmaceuticals (ATC) |
| --- | --- | --- | --- |
| Prostate specific antigen test | - | 32351 | - |
| Prostate biopsy | - | 26341 | - |
| Radical prostatectomy | 5-604 | 36276, 36277, 36287 | - |
| External-beam radiotherapy | 8-520, 8-521, 8-522, 8-523 | 25321 | - |
| Chemotherapy | 8-54 | 86512, 86514 | - |
| Brachytherapy | 8-524, 8-525 | 25333 | - |
| Orchiectomy | 5-622 | - | - |
| Hormone deprivation therapy | - | - | L02AE |
